# Alberta’s Oil Sands: Hard Evidence, Missing Data, New Promises

**DOI:** 10.1289/ehp.119-a126

**Published:** 2011-03

**Authors:** Bob Weinhold

**Affiliations:** **Bob Weinhold**, MA, has covered environmental health issues for numerous outlets since 1996. He is a member of the Society of Environmental Journalists

Pitched battles are a regular occurrence in northern Alberta, Canada, as development of the province’s oil sands continues to expand. One ongoing battle—with another salvo launched in February 2011 with the leak of a European Commission report^1^—concerns how dirty oil sands are, relative to other fuels. Another concerns the influence of the oil sands industry in monitoring its own activity.^2^ In an effort to cut through the rhetoric of health advocates, industry representatives, environmentalists, government officials, and local residents, the Royal Society of Canada (RSC) selected and covered expenses for an expert panel to winnow out the facts.

In a report issued 15 December 2010^3^ the panel cited substantial evidence that efforts to extract oil from the Alberta deposits have degraded air, land, and water quality to varying degrees. The extent of the degradation is sometimes controversial; water quality data, in particular, are subject to differing interpretations and attributions of causality. However, the panel says that, based on publicly available evidence, there appear to be no significant human health threats to the general population either now or from development anticipated in the next decade or so.

But the panel also warns that their conclusions come with a major caveat: there are major gaps in health and environmental data, risk assessments, government oversight, information transparency, industry efforts, and disaster preparedness. The health of the region could hinge on these gaps being addressed, particularly since, according to Travis Davies, a spokesman for the Canadian Association of Petroleum Producers, 97% of projected oil extraction and processing is still to come.

After the RSC panel reviewed reams of publicly available information on factors such as health status, air and water pollution, greenhouse gas emissions, land disturbance, and energy and water consumption, it concluded that “[t]he claim by some critics of the oil sands industry that it is the most environmentally destructive project on earth is not supported by the evidence. However, for Canada and Alberta, the oil sands industry involves major environmental issues on many fronts which must be addressed as a high priority.”^3p293^

## Digging and Drilling

Sprawling across much of northern Alberta’s boreal forest under an area a little smaller than the U.S. state of Illinois lies a valuable blend of bitumen, sand, minerals, and other materials.^4^ For centuries, native peoples^5^ valued the tarry blend for repairing canoes. Today, improving technology has made it possible to extract the bitumen and process it into products similar to those produced from crude oil. With today’s technology, about 27 billion m^3^—or around 10%—of the estimated bitumen deposits can be economically extracted.^4^

That puts Canada’s oil reserves second only to Saudi Arabia’s 42 billion m^3^ and a little ahead of Iran’s 23 billion.^6^ By 2025, bitumen extraction is expected to rise 2.3 times over 2010 activity.^7^ No one is willing to hazard a guess about peak activity timing or magnitude because investments are driven by unpredictable factors such as world oil prices, future technological advances, government regulation, development of alternative energy sources, and world events such as terrorism and climate change.

Extracting oil from the sands is expensive, but the 40 or so companies working the fields are finding it lucrative, with net profits of $22.8 billion in 2008.^3p3^ Preprofit expenses include payments to the Government of Alberta: $3.8 billion in 2008 alone compared with $11.9 billion over the preceding 10 years.^3p3^ Alberta has had a financial stake in the oil sands for about 80 years, since the Canadian federal government transferred ownership of most natural resources to their respective provinces.^3p17^

Surface mining is the only feasible process for extracting bitumen deposits down to a depth of 75 m. These are spread under about 4,800 km^2^ of the Athabasca oil sands region, or 3.3% of the 142,000-km^2^ bitumen-bearing zone, and account for about 20% of the estimated reserves and about 55% of current bitumen extraction.^8^,^9^ When deposits are deeper than 150 m, companies drill down and use steam heat to liberate the bitumen, a process known as *in situ* extraction. By 2015, *in situ* extraction is expected to dominate bitumen production, according to Davies.

Each process has its environmental tradeoffs. As of March 2009, surface mining had already disturbed more than 602 km^2^ of land^8^ and led to the creation of about 130 km^2^ of tailings ponds that contain dozens of toxic substances.^10^,^11^ Surface mining also requires 4–6 times more fresh water withdrawal than *in situ* extraction.^3p51^ *In situ* extraction, on the other hand, has a carbon footprint about onethird greater than that of surface mining. Both processes involve “enormous land disturbance and reclamation issues that encompass . . . the scarred landscape left by surface mines and the forest clearing that is characteristic of *in situ* production.”^3p29^ All these effects are particularly relevant to the First Nations peoples whose reserves (traditional hunting grounds) are located on or near the oil sands deposits.

Although the RSC panel found no evidence that people are currently being harmed by oil sands activity, that conclusion is based on testing for only a limited number of substances and reliance on some standards that may not be fully protective, says Kevin Timoney, an ecologist and principal investigator with Alberta-based Treeline Ecological Research. Moreover, chronic effects cannot yet be ruled out, and any health impacts later attributed to oil sands development could potentially affect tens of thousands of people living and working in and near the deposits.^3p200^

## Uncertain Impacts

There are more than 1,400 known pollutants emitted by oil sands operations.^3p222^ Among the few that are monitored are sulfur oxides (SO_X_), nitrogen oxides (NO_X_), hydrocarbons, and fine particulate matter (PM_2.5_).^12^ Emissions of SO_X_ and other sulfur compounds, NO_X_, and total hydrocarbons have been rising during the past decade, but the RSC panel concluded that “current ambient air quality monitoring data for the region show minimal air quality impacts from oil sands development . . . except for noxious odour emission problems over the past two years.”^3p281^

Indeed, hydrogen sulfide at three monitored industrial sites has exceeded the 1-hour guideline^13^ more than 2,400 times across three locations during the past decade and exceeded the 24-hour standard more than 400 times in the same period.^3p105^ Data on exceedances of the hydrogen sulfide guideline were not available for Fort McKay, a small village 54 km north of the boom town of Fort McMurray, but the RSC authors conclude that there are serious odor problems in this and possibly other locations: “Resolution of the odour problems being caused by oil sands development is clearly necessary.”^3p227^

Alberta Environment spokeswoman Jessica Potter says her agency expects industry to solve the problem. “We put in effect an environmental protection order [EPO]^14^ to ensure this happens,” she says. “EPOs are enforceable by law, and disregarding an EPO can result in criminal charges.” Meanwhile, Davies says his members are working on the issue. “It’s a learn-as-you-go scenario,” he says. “We’re trying to find different ways to fix it.”

Annual average concentrations of SO_X_, NO_X_, PM_2.5_, and carbon monoxide (CO) from 2001 to 2008 in Fort McMurray were about one-third to three-fourths the concentrations in Alberta’s major urban areas of Edmonton and Calgary,^3p95–97^ although Fort McMurray exceeded provincial 24-hour average PM_2.5_ allowances 12 times compared with Edmonton’s 9.^3p97^ PM_2.5_ exceedances at Fort McKay have been more than double those at the village of Anzac, located in the middle of traditional oil and gas operations, although the exceedances cannot be directly attributed to oil sands operations since other activities are occurring in each area. As anecdotal evidence of potential particulate matter concerns, a panel commissioned by Environment Canada to evaluate the impacts of oil sands operations referred to the “ubiquitous dust” that was present during their site visits.^15^ Findings in two small air pollutant personal exposure studies involving participants wearing portable monitors in four regional communities demonstrated indoor air provided higher contaminant exposures than ambient air.^16^,^17^

Total industry-estimated volumes of SO_X_, NO_X_, PM_2.5_, CO, volatile organic compounds, polycyclic aromatic hydrocarbons (PAHs), lead, mercury, and cadmium put the oil sands industry in anywhere from third to twelfth place—depending on the pollutant—among all Canadian industrial sources.^3p102^

Downwind from oil sands operations, elevated NO_X_ concentrations that can contribute to aquatic acidification have been detected at least 150 km east of the Alberta– Saskatchewan border, but elevated SO_X_ from oil sands was not detected at any location in Saskatchewan. One study found elevated concentrations of polycyclic aromatic compounds (PACs) in snowmelt within 50 km of oil sands operations.^18^ Despite reductions in emissions per barrel of bitumen produced, Hrudey says greenhouse gas emissions from oil sands production are about 5% of Canada’s total and are expected to continue rising because of production increases that outstrip efficiency gains.

Water pollution can potentially occur via many pathways. Massive tailings ponds associated with surface mining contain numerous toxic contaminants, including naphthenic acids, polar and saturated hydrocarbons, asphaltenes, benzene, phenols, cresols, phthalates, toluene, lead, mercury, arsenic, nickel, vanadium, chromium, and selenium.^3p124^ These can leach at low concentrations through dams and dikes, and although seepage rates must be quantified, the RSC panel notes that “very few published data are available on the dynamics of groundwater flow and the fate of process water contaminants in the impoundment structure.”^3p122^ Volatile contaminants can be transported by air, and if a tailings impoundment were to rupture, local wetlands and waterways would face a catastrophic influx of contaminants.^3p39^

*In situ* extraction processes, which use steam heated to more than 250°C, can alter subsurface dynamics such as leaching of arsenic into groundwater.^3p141^ Deep-well injection of wastes can increase the potential for groundwater and surface water contamination. Groundwater withdrawals have lowered the water table at least 40 m in some locations, altering the flows between surface water and groundwater.^19^ Overall, the RSC panel concludes, the complex interactions between surface and subsurface waters are poorly understood.

The Athabasca River is the largest single source for water for the oil sands industry, and maximum allowable water use that could occur would consume 16% of the historical 7-day low river flow. Under the current water management framework for the Athabasca River, oil sands facilities are allocated 3.5% of the average annual river flow and use less than 1%.^20^ River flow has fallen about 25–30% since the mid-1970s as precipitation declined and industrial uses increased.^21^ There are few financial incentives to reduce water use,^3p275^ but Hrudey says the RSC deemed the regulatory mechanisms in place capable of managing this issue.

Studies have found that many toxics, such as PACs, antimony, arsenic, cadmium, chromium, copper, lead, mercury, selenium, and zinc, can occur at higher concentrations downstream of oil sands operations than upstream (in some cases all the way to Lake Athabasca), and some of these are elevated enough to kill fish.^3p147^ But it remains to be determined if oil sands operations are the primary cause of these higher levels. Concentrations of toxic metals measured in the Athabasca River downstream of oil sands plants were much lower than Canadian requirements for drinking water.^22^

## Preparing for the Worst

Toxicity threats could become a major concern if there is a technological or natural disaster. A wide range of process accidents have already occurred, including numerous spills from processing plants and pipelines,^23^ fires and explosions at facilities, fires on wastewater ponds, and the deaths of more than 2,000 waterfowl that landed on various tailings ponds in multiple incidents.^3p129^

Companies are required by law to submit environmental impact assessments (EIAs) that include plans for dealing with disasters, Davies says. But those paper plans don’t always reflect a comprehensive analysis of what could go wrong, considering actual past events, according to the RSC authors: “There have been large gaps in information submitted in EIAs that have not been required by the government nor provided by the companies, specifically dealing with consequences of technological disasters.”^3p234^ They also note that EIAs rarely address how a company will deal with extreme weather such as floods, torrential rains, high winds, bitter cold, and droughts and related forest fires.^3p235^

Annual performance reports (assessments of the performance of oil sands tailings dams during operations and construction), independent dam safety reviews (which are required every five years), and emergency preparedness plans (descriptions of actions to be taken in the event a dam fails) are all available to the public through Alberta’s Freedom of Information and Protection of Privacy (FOIP) Act. Potter says emergency response plans (i.e., call-down lists or telephone trees) are the only documents that are not publicly available.

But Hrudey says information like this should be much more readily available to the public. Gillian McEachern of the advocacy group Environmental Defence explains that being forced to rely solely on the FOIP process for public access can keep quite a bit of information effectively out of reach. She says when her organization has used FOIP at the provincial or federal level, they have at times been denied, experienced lengthy delays far past the required release date, or received documents that were extensively redacted, limiting their usefulness.

Potential disasters aside, the combined effects of boom-town development and deficits in health care infrastructure are among the suspects in a number of poorer population health indicators for the region encompassing oil sands development compared with other similarly remote and low-density regions of Alberta.^3p288^ That pattern indicates to the RSC panel that efforts to improve the situation are essential: “There are obvious health indicator disparities that are not acceptable in a region that is generating so much wealth for the province and the country, and these disparities need to be addressed regardless of their cause.”^3p238^

André Corriveau, chief medical officer of health for Alberta Health and Wellness, says his agency has been working with other stakeholders on plans for a comprehensive health assessment, including biomonitoring and air and water quality monitoring, for the community of Fort Chipewyan. That small town was the setting for a controversial cancer cluster claim in the mid-2000s. The RSC report concludes that, based on the available evidence, there are no convincing data showing a link between cancers at Fort Chipewyan and oil sands operations but that additional monitoring and research are warranted.^3p227^

Corriveau says he is waiting on the outcome of pending community consultations so new work can begin there. He also says he has had preliminary discussions with oil sands company representatives regarding access to internal data on current health monitoring of workers that could be relevant for analysis of potential occupational health impacts. No other health studies specific to oil sands are in the works, he says.

One of the primary issues in conducting such studies, and in the RSC panel’s conclusion of no serious current health problems caused by oil sands operations, is the inadequacy of some current health standards, Timoney says. For instance, he says Health Canada’s guideline for mercury in fish is much higher than that of the U.S. Environmental Protection Agency, and there are no guidelines for important pollutants such as PAHs in sediment that can get into fish and drinking water. In addition, he says that “standards and guidelines are only useful if they are followed and enforced. Enforcement is the exception rather than the rule.”

## A Shift in Direction?

Existing laws provide clear guidance to the Government of Alberta about how to adequately manage environmental impacts of oil sands development, concludes the RSC panel: “[T]he government simply needs to respect the letter of its own legislation.”^3p290^ But that’s been difficult, as provincial and federal agencies—even with overlapping jurisdictions, responsibilities, and agreements— have not always demonstrated optimal regulatory capacity or management.^3p78^

Those struggles continue today and were one reason why the RSC panel determined “there was little evidence available to us that implementation of meaningful improvements has begun or will be achieved in an adequately rapid time frame.”^3p295^

Many agencies and organizations have monitoring systems in place in an effort to track certain health and environmental impacts related to oil sands development. But a decade after bitumen extraction began to rapidly increase, the overall system remains weak, according to the panel of experts commissioned by Environment Canada.^15^ That panel, which released its report a week after the RSC’s, says the current monitoring program is “dwarfed by the level of activity that was expended on other major environmental issues of the past few decades, such as the acid deposition problem in eastern Canada,”^15p30^ and that it is riddled with inadequacies, including poor leadership, a lack of clear objectives, weak decisionmaking processes, poor project design, and fragmented and incomplete systems, projects, and data.

Some of the sharpest criticism was directed toward the industryfunded Regional Aquatics Monitoring Program (RAMP), whose members include industry, government, and First Nations organizations. RAMP was formed in 1997 in accordance with recommendations by Environment Canada to monitor the health of water bodies in the oil sands region. Along with the problems noted above, the Environment Canada authors say RAMP has poor scientific leadership and data transparency and fails to communicate well with scientists and the public.^15^ RAMP officials say they are reviewing the Environment Canada and RSC reports, as well as that of a provincially commissioned scientific panel that was released in January 2011, which, consistent with the other reports, recommended extensive changes to the current program.^24^

The Environment Canada panelists echoed the findings of the RSC authors when they wrote, “[W]ork carried out to date has not led to a consensus on the degree of impacts [of oil sands development]. . . . However, there is not a widespread scientific acceptance of this negative finding because of the lack of complete confidence in the monitoring system that produced the result.”^15p31^ The panel wrapped up its report with a series of conceptual recommendations for an improved monitoring system and said industry should pay any new costs.^15p42^ Davies of the Canadian Association of Petroleum Producers says association members “are willing to pay for a worldclass system. I think it’s in our best interest.”

Several agencies and organizations say changes are on the way. Alberta Environment officials acknowledge they have done little to assess or address cumulative impacts of oil sands development, but are beginning to evaluate how to do so. Potter says they are looking at how to improve the financial security structure so the oil sands industry is held accountable for failures and disasters.

Another effort in process, with full implementation expected by 2012, is a revision of reclamation requirements and oversight at the national and provincial level. In addition, officials at Alberta Environment are working collaboratively with the federal government to examine the technical aspects of a physical network for water monitoring in the oil sands region. The federal panel’s recommendations for water monitoring are expected in March 2011. Alberta will incorporate those findings into its own review, expected by June 2011. “The federal government panel is only looking at technical aspects of a physical network for water monitoring in the oil sands region; the provincial panel will include the necessary governance, reporting, validation, and analyses portions,” Potter says. “This will be expanded to air, land, and biodiversity not just for the oil sands but across the province.”

The day after the Environment Canada panel report was released, RAMP posted online data related to topics such as water quality, sediment quality, aquatic vegetation, benthic invertebrates, and acid-sensitive lakes.^25^ The newly released database does not include groundwater monitoring reports, Price says, although his organization has sometimes been able to acquire such reports after hiring investigators to scour government libraries. The industry has also improved in areas such as tailings pond management and energy efficiency,^3p40^ and Davies says “the onus is on the industry to continue to improve their performance.”

Such a commitment is welcomed by Terra Simieritsch, senior policy analyst with the Pembina Institute, a Canadian environmental think tank. “There are many changes required in order to shift oil sands development towards a path where it is being done responsibly,” she says. “We’ve seen a lot of denial of the impacts. There’s been a lot of focus on public relations, whereas the focus should be on what is actually happening on the ground.” When asked if she expects changes that are beneficial from her perspective to occur, she’s hesitant, but leaves the door open: “It remains to be determined.”

## Figures and Tables

**Figure:**
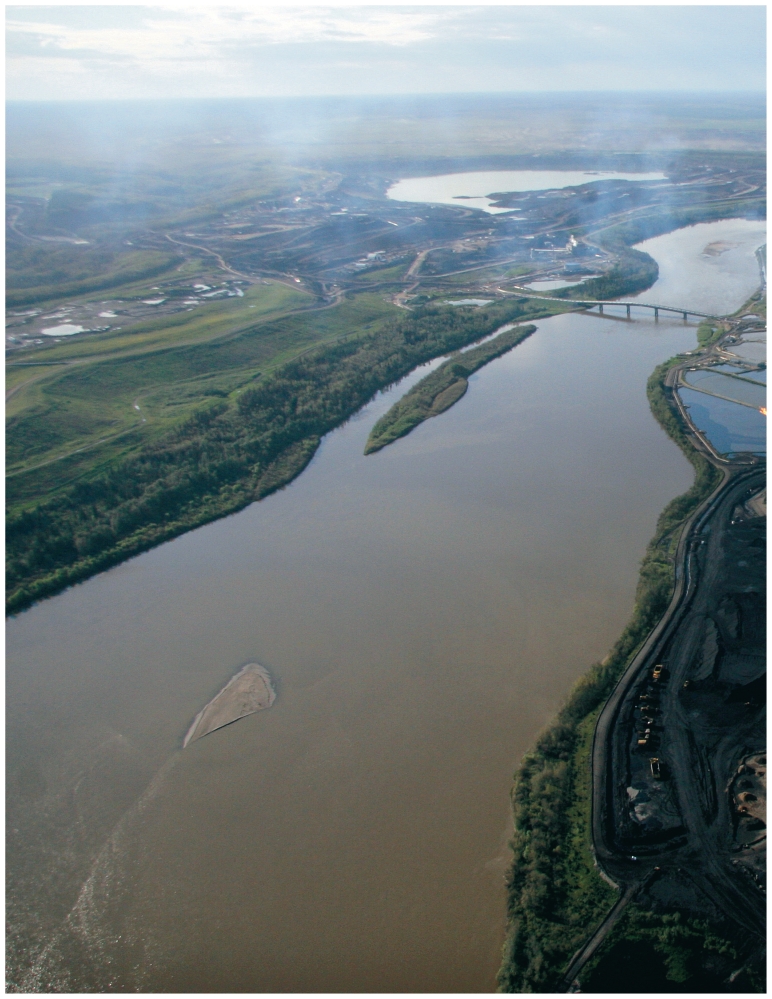

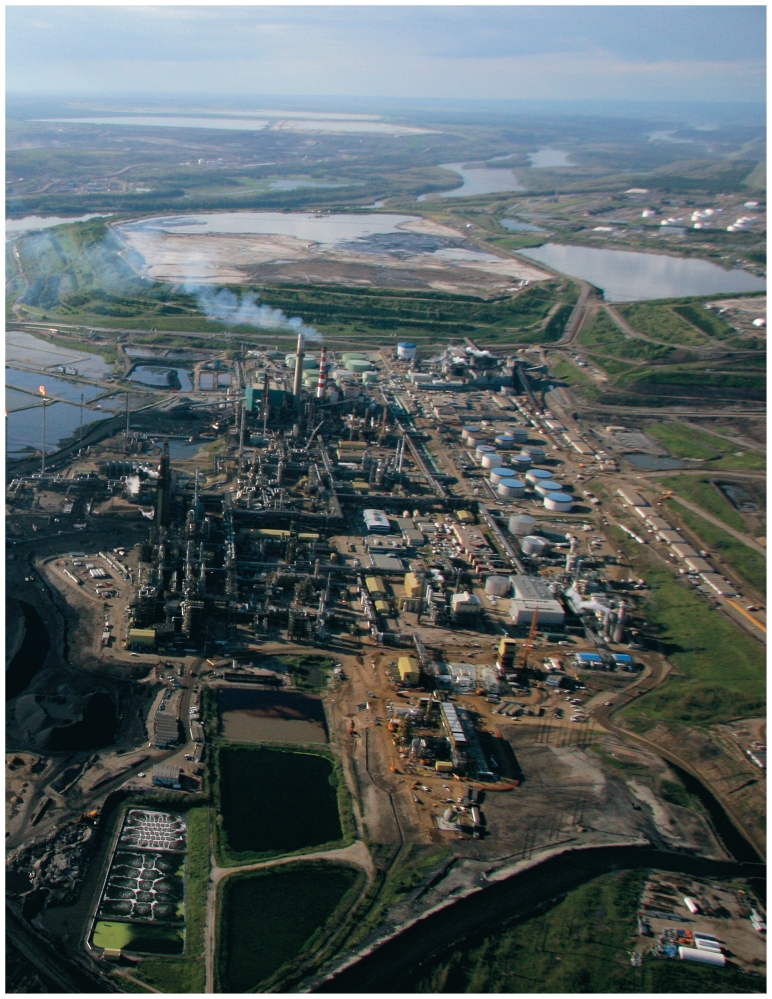
The Athabasca River runs by Suncor Energy’s oil sands upgrader facility near Fort McMurray. Fort Chipewyan lies about 280 km upstream.

**Figure:**
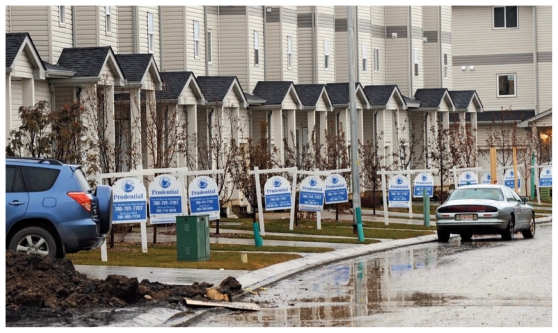
In late 2009 these houses were selling for around US$450,000 at a new property development in Fort McMurray, a boom town about 30 km south of the nearest oil sands operations. Transient workers with the oil sands and other industries make population estimates tricky, but by one estimate the population of Fort McMurray grew by 80% between 2000 and 2010, reaching nearly 77,000; it is expected to nearly double again by 2028.^26^

**Figure:**
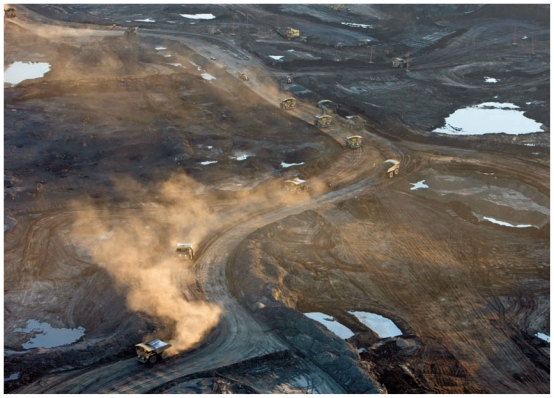
Suncor Millennium Mine, north of Fort McMurray. In its December 2010 report, an expert panel commissioned by Environment Canada to assess oil sands monitoring research wrote, “[O]ur site visits had an indelible impact. It is hard to forget the sheer extent of landscape disruption, the coke piles and the ubiquitous dust.”^15^

**Figure:**
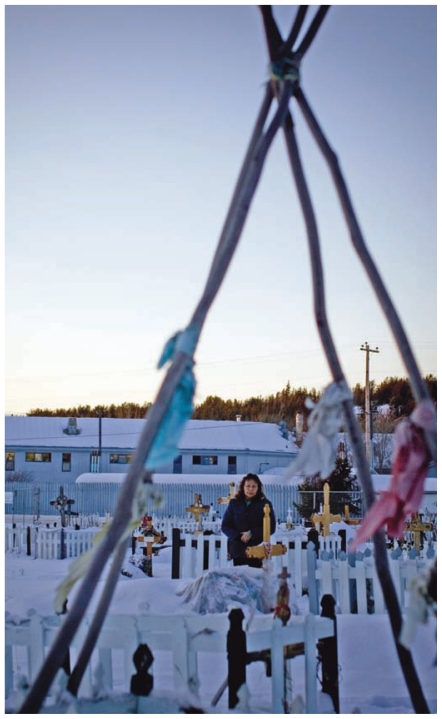
The RSC panel found that the available evidence did not support a link between cancers at Fort Chipewyan and oil sands operations, although additional monitoring and research are warranted. That leaves this Fort Chipewyan woman still uncertain over what caused the lung cancer that killed her mother, husband, and 27-year-old nephew between 2006 and 2008.
